# Moderate calorie restriction to achieve normal weight reverses β-cell dysfunction in diet-induced obese mice: involvement of autophagy

**DOI:** 10.1186/s12986-015-0028-z

**Published:** 2015-10-06

**Authors:** Xiuying Gao, Dien Yan, Yinan Zhao, Hong Tao, Yingsheng Zhou

**Affiliations:** Department of Endocrinology and Metabolism, Beijing Anzhen Hospital, Capital Medical University, Beijing, 100029 China; Beijing Institute of Heart, Lung, and Blood Vessel Diseases, Beijing, 100029 China

**Keywords:** Calorie restriction, β-cell function, Autophagy

## Abstract

**Background:**

Severe calorie restriction (CR) is shown to improve or even reverse β-cell dysfunction in patients with obesity and type 2 diabetes mellitus. However, whether mild to moderate CR can reverse β-cell dysfunction induced by obesity and the underlying mechanism remain unclear. Autophagy plays an important role in maintaining mass, architecture and function of β-cells. While the impact of CR on β-cell autophagy is unknown. This study aims to investigate the effects of moderate CR on β-cell function and autophagy activity in diet-induced obese (DIO) mice.

**Methods:**

DIO C57BL/6 mice were subjected to 3 weeks of switching to normal chow (HF → NC group) or normal chow with 40 % CR (HF → NC CR group). Then hematoxylin-eosin and immunohistochemistry staining were performed to observe β-cell morphology. β-cell function was evaluated by intraperitoneal glucose tolerance test in vivo and static GSIS (glucose-stimulated insulin secretion) in isolated islets. β-cell autophagy activity was determined by transmission electron microscope and western blot.

**Results:**

In the HF → NC CR group, CR normalized body weights, completely restored glucose tolerance, early-phase and second-phase insulin secretion, insulin sensitivity, and islet size. CR also normalized insulin content and glucose-stimulated insulin secretion in isolated islets *in vitro*. Furthermore, β-cell autophagy level was increased in the HF → NC CR group, but AMPK phosphorylation remained unchanged. Although HF → NC mice achieved moderate weight loss and normal glucose tolerance, their insulin secretion was not improved compared with obese control mice, and additionally, β-cell autophagy was not activated in these mice.

**Conclusions:**

Moderate (40 %) CR to achieve normal weight reversed β-cell dysfunction and insulin resistance, and restored glucose homeostasis in DIO mice. Furthermore, the up-regulation of β-cell autophagy may play a role in this process, independent of AMPK activation.

## Background

The prevalence of obesity and associated health consequences, including type 2 diabetes mellitus (T2DM), continues to increase [1, 2]. Obesity not only causes insulin resistance, but also leads to pancreatic β-cell damage which manifests as the impaired insulin secretion in response to glucose and nonfuel stimuli rather than reduced β-cell mass [3]. Diet control can effectively reduce body weight and delay the progression of obesity to T2DM [4]. However, the method of dietary intervention has not been standardized, and it is important to find an effective and feasible dietary intervention strategy to improve or even reverse obesity-induced β-cell dysfunction.

Calorie or dietary restriction (CR) can significantly improve β-cell function in subjects with obesity and T2DM. Clinical studies have demonstrated an improvement in insulin secretion in morbidly obese patients with T2DM, after 1 week of very low–calorie diet (VLCD) of 400 kcal/day or 3 weeks of 500 kcal/day [5, 6]. Furthermore, another study have indicated that insulin secretion rate and first-phase insulin response are normalized after 8 weeks of VLCD (600 kcal/day) in patients with T2DM [7]. All these clinical studies apply severe CR, which is difficult to be accepted by most patients. However, the effects of mild-to-moderate CR on β-cell function in subjects with obesity have not been well investigated, and related studies mainly focus on the improvement of insulin resistance [8, 9]. Studies of CR in obese animal models often use 30–40 % (moderate) CR with a focus on insulin resistance, however, the effect of CR on β-cells has not been studied extensively [10, 11]. Therefore, whether mild-to-moderate CR can reverse β-cell dysfunction induced by obesity has not yet been demonstrated and the molecular mechanisms mediating this beneficial effect remain unclear.

Autophagy as a cellular degradation-recycling system for aggregated proteins and damaged organelles can be induced under energy deprivation conditions such as starvation [12, 13]. Autophagy plays an important role in maintaining mass, architecture and function of β-cells [14]. A study has shown an increased β-cell autophagy in high-fat-fed mice, and inductive β-cell autophagy acts as a compensatory mechanism in response to obesity [13]. However, the impact of weight loss or CR on β-cell autophagy remains unknown.

This study used diet-induced obese (DIO) mice subjected to 3 weeks of switching to normal chow (HF → NC) or normal chow with 40 % CR (HF → NC CR), and then investigated whether moderate (40 %) CR could reverse β-cell dysfunction induced by obesity. Furthermore, we also sought to explore whether β-cell autophagy was involved in this process.

## Methods

### Animals and diets

**Fig. 1:**
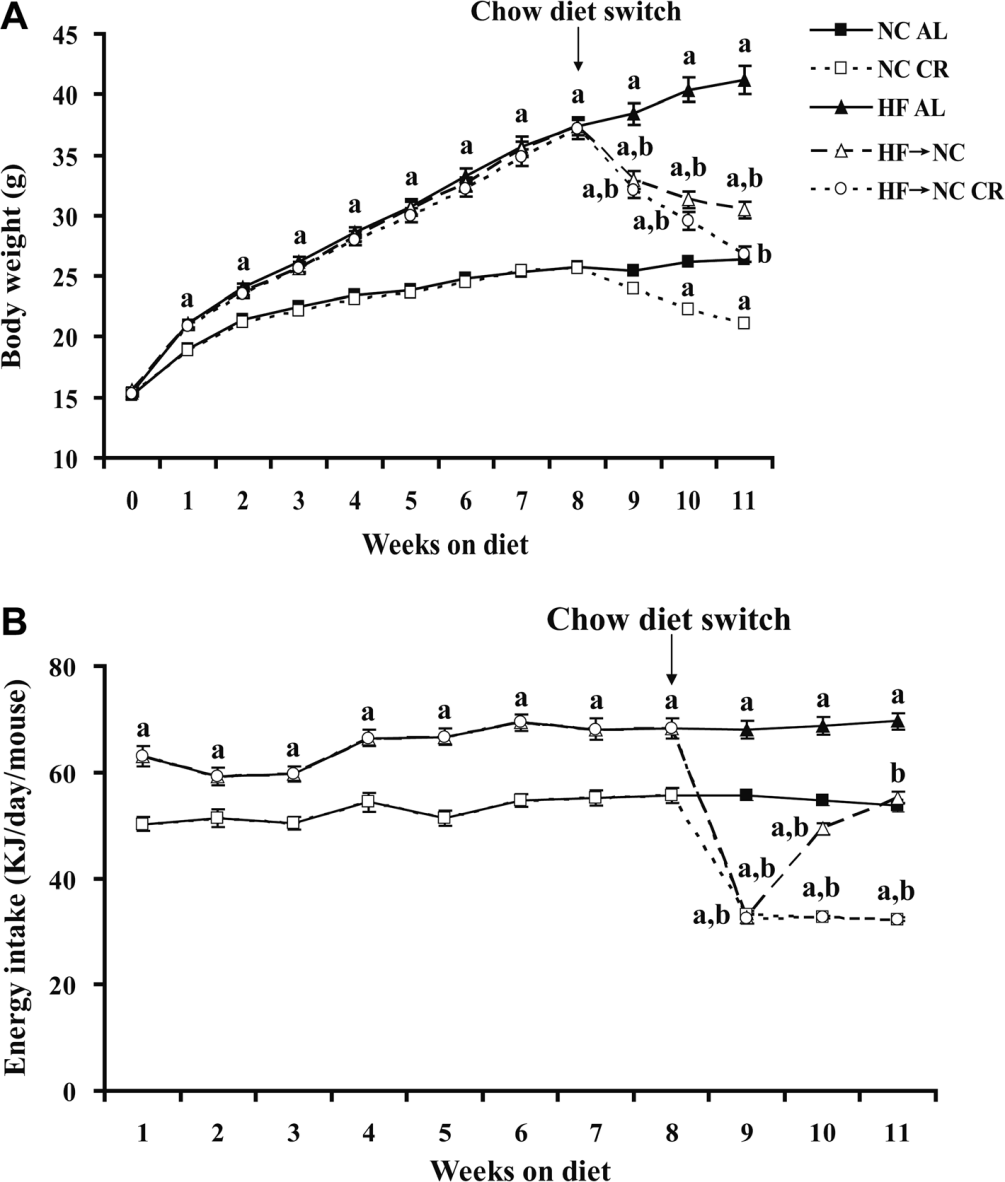
The body weight and energy intake of the five groups during the experiment. Male C57BL/6 mice were fed either NC or HF for 8 weeks beginning at age 4 weeks, then they were divided into five groups: NC AL, NC CR (40 % CR), HF AL, HF → NC, HF → NC CR (40 % CR), these treatments continued for another 3 weeks. **a** the body weight and (**b**) energy intake were evaluated throughout the experimental period. Data were presented as means ± SE, *n* = 20. *a* indicates *P* < 0.05 versus NC AL group; *b* indicates *P* < 0.05 versus HF AL group. Abbreviations: NC, normal chow; HF, high-fat diet; AL, ad libitum; CR, calorie restriction

Four weeks old male C57BL/6 mice were purchased from Vital River Laboratories (Beijing, CHN). They were random fed high-fat diet (HF) (Research Diets D12492; 60 % KJ from fat, 20 % KJ from carbohydrate, and 20 % KJ from protein, 21.9 KJ/g, *n* = 60) or normal chow (NC) (Research Diets D12450J; 10 % KJ from fat, 70 % KJ from carbohydrate, and 20 % KJ from protein, 16.1 KJ/g, *n* = 40) for 8 weeks. Then they were randomly divided into the following five groups (*n* = 20/group): NC AL (ad libitum), NC CR, HF AL, HF → NC (HF switching to NC, ad libitum), HF → NC CR. The food intakes of both CR groups were 60 % of that of NC AL group (Fig. 1b). These treatments continued for 3 weeks, because at that time body weights of HF → NC CR mice reduced to the same level as NC AL mice (Fig. 1a). Food intake and body weight were measured on a daily and weekly basis, respectively. All procedures were approved by the Animal Ethics Commission of Capital Medical University.

### Metabolic and plasma parameters

After overnight fasting and anesthetization, the body length, body weight, and abdominal circumference were measured, and then Lee index was calculated [15]. Besides, epididymal fat, liver and pancreas were harvested and weighted. Plasma triglycerides, total cholesterol, non-esterified fatty acids (NEFA) and adiponectin concentrations were determined by commercially available kits (Wako for lipid profiles; Alpco for adiponectin).

### Intraperitoneal glucose tolerance test (IPGTT)

After a 16 h fasting, 25 % glucose (2 g/kg) was administered intraperitoneally. Tail blood samples were collected at 0, 15, 30, 60, and 120 min, and blood glucose was measured by a portable glucometer (Contour^TM^TS). Plasma insulin level was measured by ELISA (Alpco). Insulinogenic index was calculated to assess early-phase insulin secretion according to the formula: [15 min insulin (μU/mL)-fasting insulin] / [15 min glucose (mmol/L)-fasting glucose] [16]. Area under the curve (AUC) for glucose and AUC for insulin (representing second-phase insulin secretion) were calculated using the trapezoid method. Insulin resistance was estimated by homeostasis model assessment of insulin resistance (HOMA-IR).

### Histological analysis

The samples of pancreas were fixed in 10 % neutralized formalin and then embedded in paraffin and cutted into 5 μm sections. Standard hematoxylin and eosin (HE) stainings were performed. The pancreas sections were subjected to immunohistochemistry (IHC) staining with primary antibody against insulin (1:400, Santa Cruz). Images were captured with a microscope (ECLIPSE80i/90i).

### Transmission electron microscopy (TEM)

After fixation in 3 % glutaraldehyde, pancreas tail samples were postfixed with 1 % OsO4 and embedded in Epon812. Ultrathin sections were mounted and stained with uranyl acetate/lead citrate (JEM-1400).

### Islet isolation

Islets were isolated from overnight fasted mice by the collagenase digestion technique and followed by overnight culture for recovery [17]. To determine islet size, images were taken using a DMI 4000B inverted microscope (Leica) and analyzed by Image-Pro Plus 3.0 software.

### Glucose-stimulated insulin secretion and insulin content measurements

Static glucose-stimulated insulin secretion (GSIS) experiment was performed as described [17]. Briefly, islets were starved in Krebs-Ringer bicarbonate-HEPES buffer (KRBH) for 1 h. Then batches of 10 size-matched islets were pipetted into 24-well plates and treated with 2 ml of KRBH containing 2.8 mM or 16.7 mM glucose for 1 h. The supernatant was collected for assay of insulin release. For determination of islet insulin contents, aliquots of five islets were lysed in acid-ethanol solution [18]. Insulin was measured by ELISA as described above.

### Western blot analysis

Groups of 300 freshly isolated islets (*n* = 6) were pelleted and resuspended in 40 μL of lysis buffer. Equal amounts of protein (30 μg) were resolved by electrophoresis and then transferred to PVDF membranes. The membranes were probed overnight at 4 °C with primary antibodies against LC3 (1:500), p62 (1:1000) (both MBL), beclin-1 (1:1000), pT172-AMPKα (1:500), and AMPKα (1:500) (all Cell Signaling), and then incubated with Dylight-labeled secondary antibodies (1:10000, KPL) for 1 h. Images were quantified by the Odyssey infrared imaging system (LI-COR Biosciences).

### Statistical analysis

Measurement data was expressed as means ± SE. One-way ANOVA with LSD or S-N-K post hoc testing were employed for multiple comparisons. Categorical variables were presented as percentages and analyzed by Chi-squared test. *P* < 0.05 was considered statistically significant.

## Results

### Body weight, organs weight and plasma parameters

As shown in Fig. 1a, after 8 weeks of HF feeding, DIO models were successfully established [19], with a 45.1 % marked increase in body weight compared with control mice, meanwhile, the energy intake was significantly increased by 22.8 % (Fig. 1b). After 3 weeks of CR intervention, HF → NC CR mice achieved normal weight. Although the HF → NC group achieved a 26.1 % weight reduction compared with HF AL mice, they remained more obese than NC AL mice.

As shown in Table 1, HF AL mice showed dramatically increased abdominal circumference and significantly increased weights of epididymal fat, liver and pancreas, along with hyperlipidemia and hypoadiponectinemia. In the HF → NC CR group, these parameters were normalized or even lower than those in the NC AL group. Although adiposity and metabolic characteristics were improved in the HF → NC group, the epididymal fat weight, blood lipid profile, and plasma adiponectin level still did not return to normal levels. Besides, NC CR group showed the lowest body weight and metabolic parameters, however, the pancreas weight, plasma total cholesterol, NEFA and adiponectin levels were unaffected by 3 weeks of CR.

**Table 1:** Metabolic characteristics of the five groups at the end of the study

Parameter	NC AL	NC CR	HF AL	HF → NC	HF → NC CR
Body weight (BW, g)	26.35 ± 0.38	21.12 ± 0.33 ^*^	41.23 ± 1.16 ^*^	30.49 ± 0.70 ^*,**^	26.83 ± 0.67 ^**^
Abdominal circumference (cm)	7.4 ± 0.2	6.6 ± 0.1 ^*^	9.0 ± 0.2 ^*^	7.6 ± 0.1 ^**^	6.9 ± 0.1 ^*,**^
Lee index	323.18 ± 2.89	313.09 ± 3.67 ^*^	332.83 ± 2.43 ^*^	321.82 ± 2.75 ^**^	320.38 ± 3.00 ^**^
Epididymal fat weight (%BW)	1.41 ± 0.11	0.20 ± 0.06 ^*^	4.94 ± 0.19 ^*^	2.37 ± 0.25 ^*,**^	1.26 ± 0.32 ^**^
Liver weight (mg)	1115.0 ± 20.0	828.0 ± 47.3 ^*^	1263.1 ± 74.1 ^*^	1052.2 ± 45.4 ^**^	882.0 ± 35.2 ^*,**^
Pancreas weight (mg)	184.0 ± 20.0	186.3 ± 9.7	295.0 ± 14.9 ^*^	211.1 ± 13.8 ^**^	194.4 ± 11.1 ^**^
Fasting blood glucose (mmol/L)	4.9 ± 1.2	3.6 ± 1.8	7.9 ± 2.2 ^*^	5.8 ± 0.7 ^**^	5.7 ± 1.5 ^**^
Fasting plasma insulin (ng/mL)	0.18 ± 0.05	0.13 ± 0.08	0.74 ± 0.14 ^*^	0.29 ± 0.07 ^*,**^	0.20 ± 0.07 ^**^
AUC_glc0–120_ (mmol/L × min)	880.1 ± 119.4	423.6 ± 109.1 ^*^	1768.3 ± 166.8 ^*^	1077.2 ± 136.5 ^**^	569.9 ± 100.0 ^**^
AUC_ins0–120_ (ng/mL × min)	22.66 ± 1.92	18.96 ± 4.22	34.86 ± 2.34 ^*^	25.74 ± 3.86 ^**^	24.54 ± 2.70 ^**^
Insulinogenic index	0.56 ± 0.07	0.96 ± 0.21	0.32 ± 0.22	0.59 ± 0.09	0.93 ± 0.22 ^**^
HOMA-IR	0.87 ± 0.12	0.58 ± 0.17	5.97 ± 1.00 ^*^	1.54 ± 0.11 ^**^	1.17 ± 0.25 ^**^
Plasma triglycerides (mg/dl)	30.28 ± 2.16	19.63 ± 2.40 ^*^	43.86 ± 3.88 ^*^	38.13 ± 2.97 ^*^	16.47 ± 1.19 ^*,**^
Plasma total cholesterol (mg/dl)	70.60 ± 3.59	58.97 ± 3.85	152.17 ± 6.65 ^*^	83.72 ± 4.60 ^*,**^	65.34 ± 3.30 ^**^
Plasma NEFA (mmol/L)	0.79 ± 0.06	0.63 ± 0.05	1.30 ± 0.06 ^*^	1.03 ± 0.10 ^*,**^	0.67 ± 0.06 ^**^
Plasma adiponectin (μg/ml)	18.94 ± 1.18	18.71 ± 3.17	11.82 ± 1.64 ^*^	12.80 ± 1.85 ^*^	17.71 ± 1.49 ^**^

Data are presented as mean ± SE, *n* = 8 ~ 16. * indicates *P* < 0.05 versus NC AL group; ** indicates *P* < 0.05 versus HF AL group

*Abbreviations*: *NC* normal chow, *HF* high-fat diet, *AL* ad libitum, *CR* calorie restriction, *NEFA* non-esterified fatty acid

### Effects of CR on pancreatic islet morphology and β-cell area

**Fig. 2:**
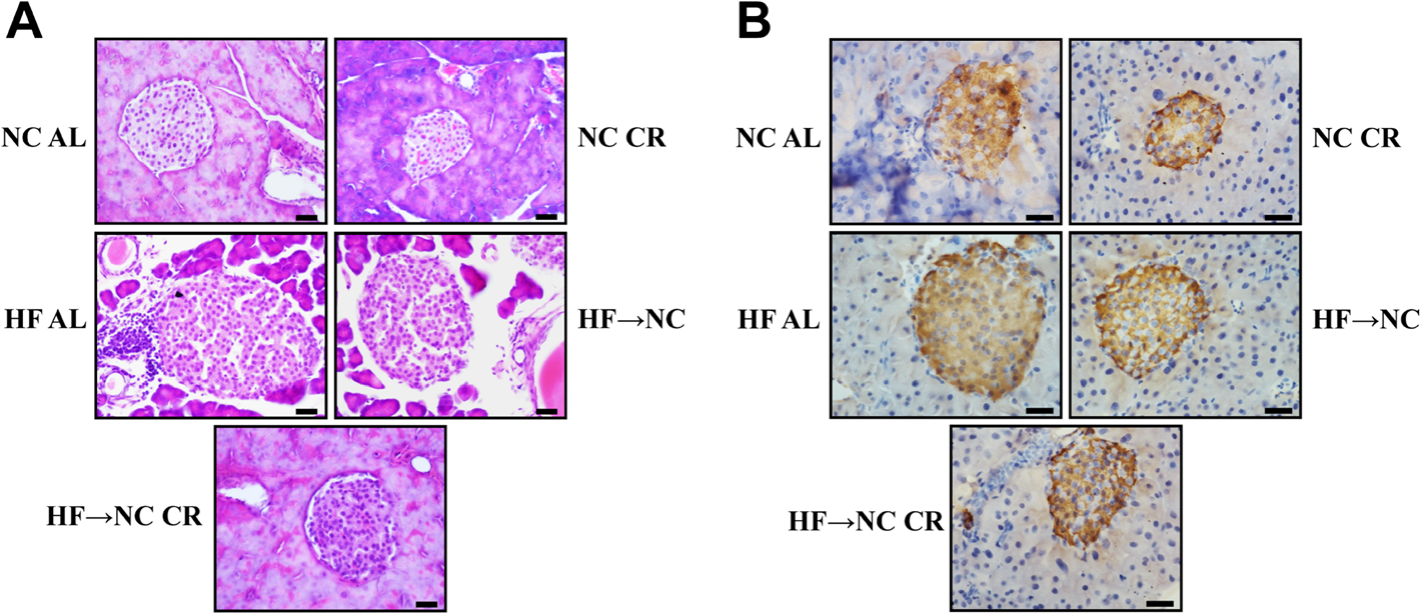
Effects of CR on pancreatic islet morphology and β-cell area. **a** representative images of H&E staining of pancreatic sections. Scale bars: 20 μm. **b** pancreatic sections were immunostained for insulin and the positive staining area represented β-cell area. Scale bars: 20 μm

**Fig. 3:**
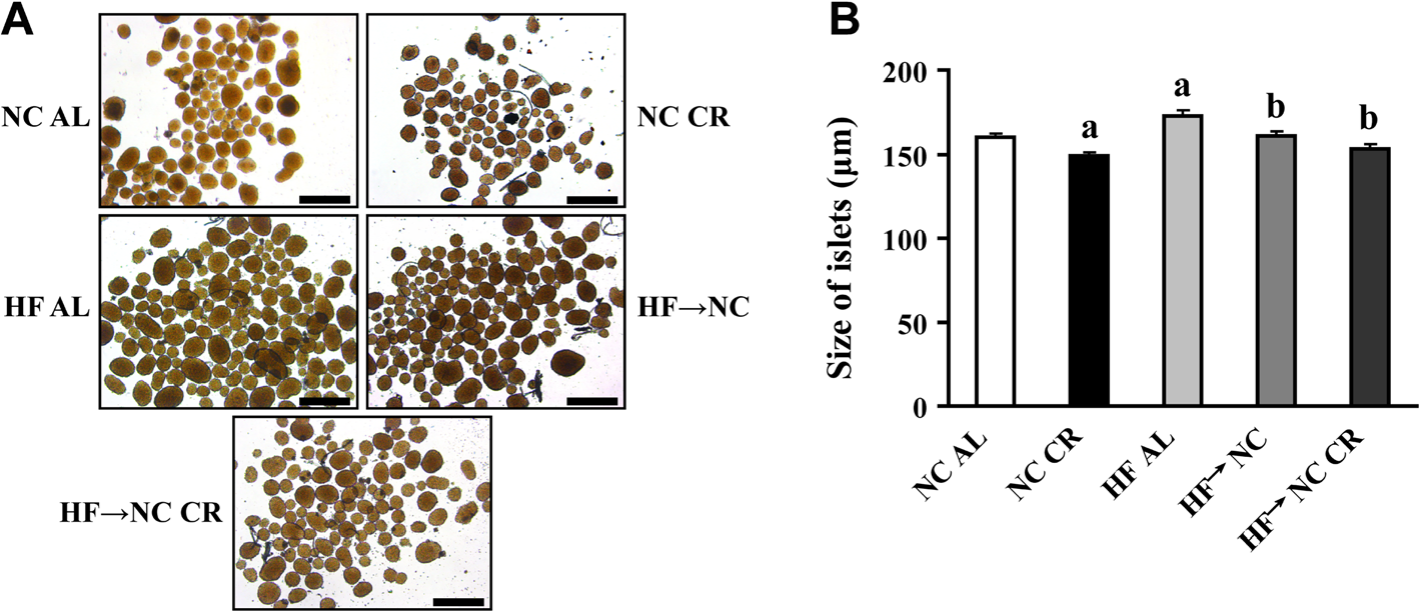
Effects of CR on the size of isolated islets. **a** representative images of isolated islets after overnight culture for recovery. Scale bars: 500 μm. **b** the mean size of islets determined by measuring of 250 ~ 350 islets (from 5 mice) per group. *a* indicates *P* < 0.05 versus NC AL group; *b* indicates *P* < 0.05 versus HF AL group

HE and IHC staining demonstrated hypertrophic islets and increased β-cell area (positive staining area for insulin) in the pancreas of HF AL mice (Fig. 2a and b), which were improved in the HF → NC mice and normalized in the HF → NC CR mice. Similar trends were also observed in isolated islets *ex vivo* (Fig. 3a). Quantitative analysis revealed that islet size was increased by 8.1 % in HF AL mice and reduced to normal size in both the HF → NC and HF → NC CR groups (Fig. 3b). Additionally, NC CR mice had the smallest islets.

### Effects of CR on glucose tolerance, insulin sensitivity, insulin secretion and insulin content

**Fig. 4:**
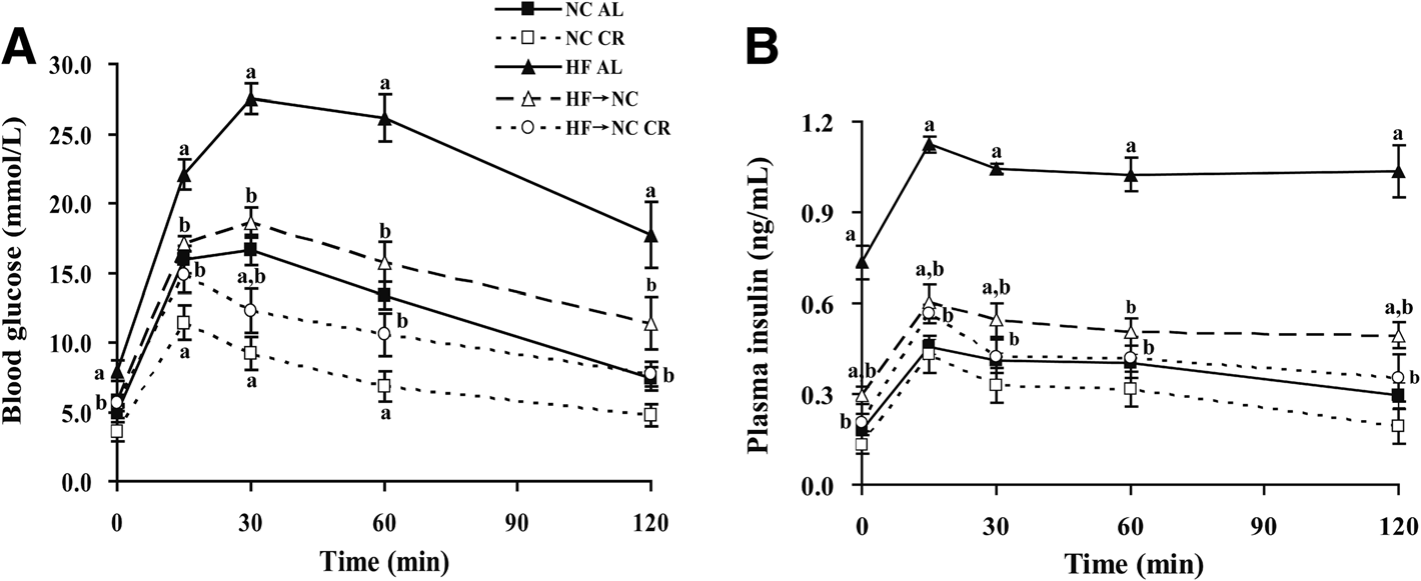
Effects of CR on glucose tolerance, early-phase and second-phase insulin secretion and insulin sensitivity. Blood glucose (**a**) and plasma insulin (**b**) were measured at times 0, 15, 30, 60, and 120 min in response to a glucose challenge (i.p. 2 g/kg) in 16-h fasted conscious mice. Data were presented as means ± SE, *n* = 6–8 mice/group. *a* indicates *P* < 0.05 versus NC AL group; *b* indicates *P* < 0.05 versus HF AL group. Abbreviations: i.p., intraperitoneally; AUC, area under the curve; HOMA-IR, homeostasis model assessment of insulin resistance

As shown in Fig. 4a, b and Table 1, HF AL mice developed glucose intolerance and insulin resistance. Fasting blood glucose, AUC for glucose, fasting plasma insulin, and HOMA-IR in HF AL mice were increased by 60.7 %, 1.0-fold, 3.1-fold, and 5.9-fold, respectively. The AUC for insulin (second-phase insulin secretion) was increased by 53.8 %, whereas the insulinogenic index (early-phase insulin secretion) tended to decrease, though no statistically different from the NC AL group (Table 1). After CR intervention, all these parameters were normalized in the HF → NC CR group. HF → NC mice showed normal glucose tolerance and higher insulin levels, implying that insulin resistance remained higher than in the NC AL group. Furthermore, they also showed unimproved early-phase insulin secretion compared to the HF AL group. NC CR mice presented normal plasma insulin levels and unchanged early-phase insulin secretion, but blood glucose levels at 15, 30 and 60 min of IPGTT were reduced compared with the NC AL group.

**Fig. 5:**
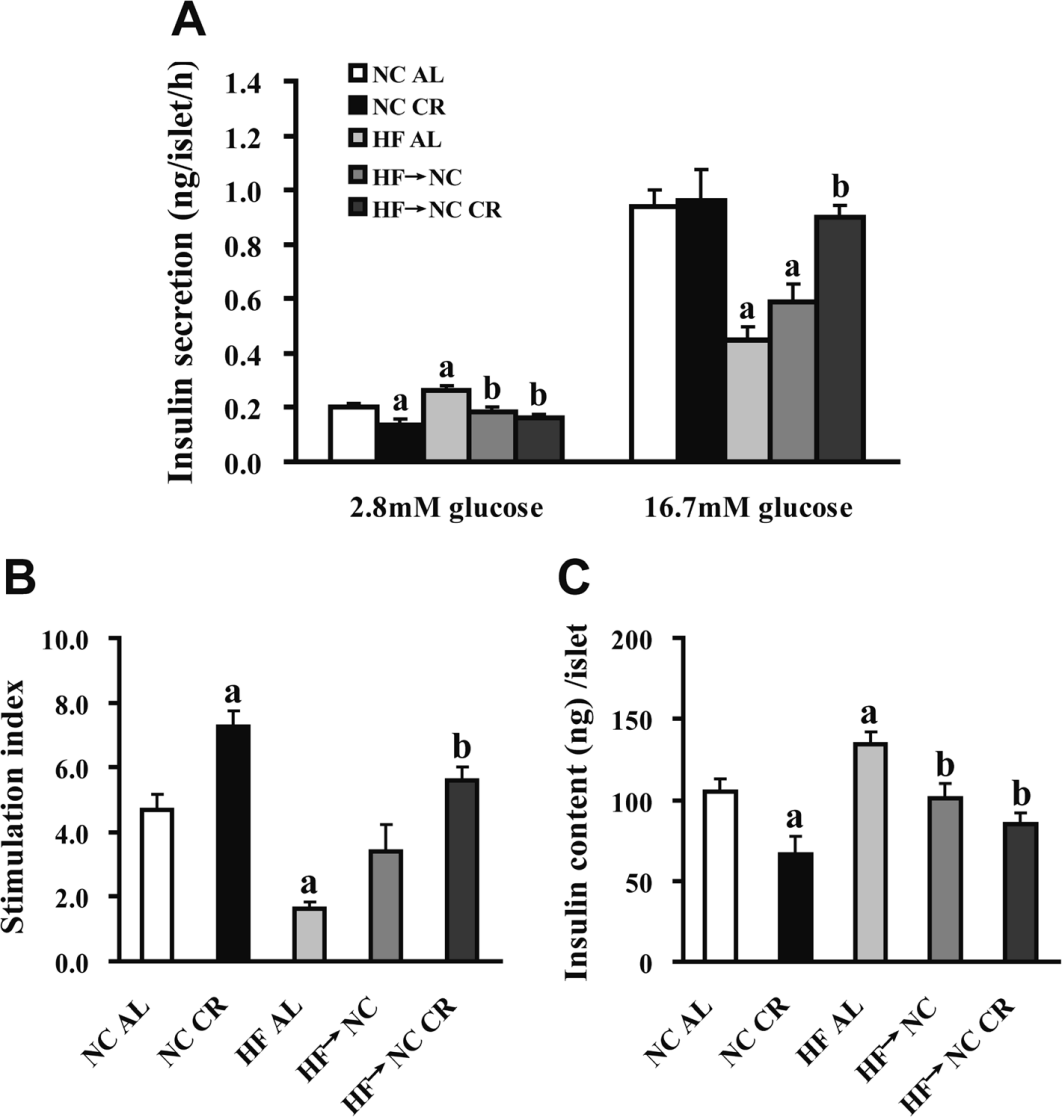
Effects of CR on glucose-stimulated insulin secretion and insulin content in isolated islets. **a** glucose-stimulated insulin secretion was measured in isolated islets (10 islets per concentration in triplicates) at 2.8 and 16.7 mM glucose. **b** the stimulation index calculated by dividing insulin secretion in response to 16.7 mM glucose by that in response to 2.8 mM glucose. **c** insulin content of isolated islets was measured in batches of 5 islets in triplicates. Data were presented as means ± SE, *n* = 5 mice/group. *a* indicates *P* < 0.05 versus NC AL group; *b* indicates *P* < 0.05 versus HF AL group

To eliminate the interference of insulin resistance *in vivo*, we evaluated GSIS in isolated islets *ex vivo*. As shown in Fig. 5a, HF AL islets had defective insulin secretion, with a 30.6 % increase in basal insulin secretion and a 52.1 % reduction of insulin secretion in response to 16.7 mM glucose compared with NC AL group. For HF → NC CR islets, both basal and high glucose-stimulated insulin secretion were completely restored. Although basal insulin secretion returned to normal in HF → NC islets, high glucose-stimulated insulin secretion was still unimproved relative to the HF AL group. The stimulation index (SI) was calculated by dividing high glucose-stimulated insulin secretion by basal insulin secretion. HF AL islets displayed the lowest SI, which was not enhanced in HF → NC islets but fully recovered in HF → NC CR islets (Fig. 5b). Insulin content in isolated islets increased by 28.3 % in the HF AL group and reduced to normal levels in both the HF → NC and HF → NC CR groups (Fig. 5c), in accordance with the results of the IHC analysis. NC CR islets showed the lowest insulin content and basal insulin secretion, however, high glucose-stimulated insulin secretion was comparable with NC AL islets.

### Effects of CR on autophagy and expression of phosphorylated adenosine monophosphate-activated protein kinase (AMPK) in islets

**Fig. 6:**
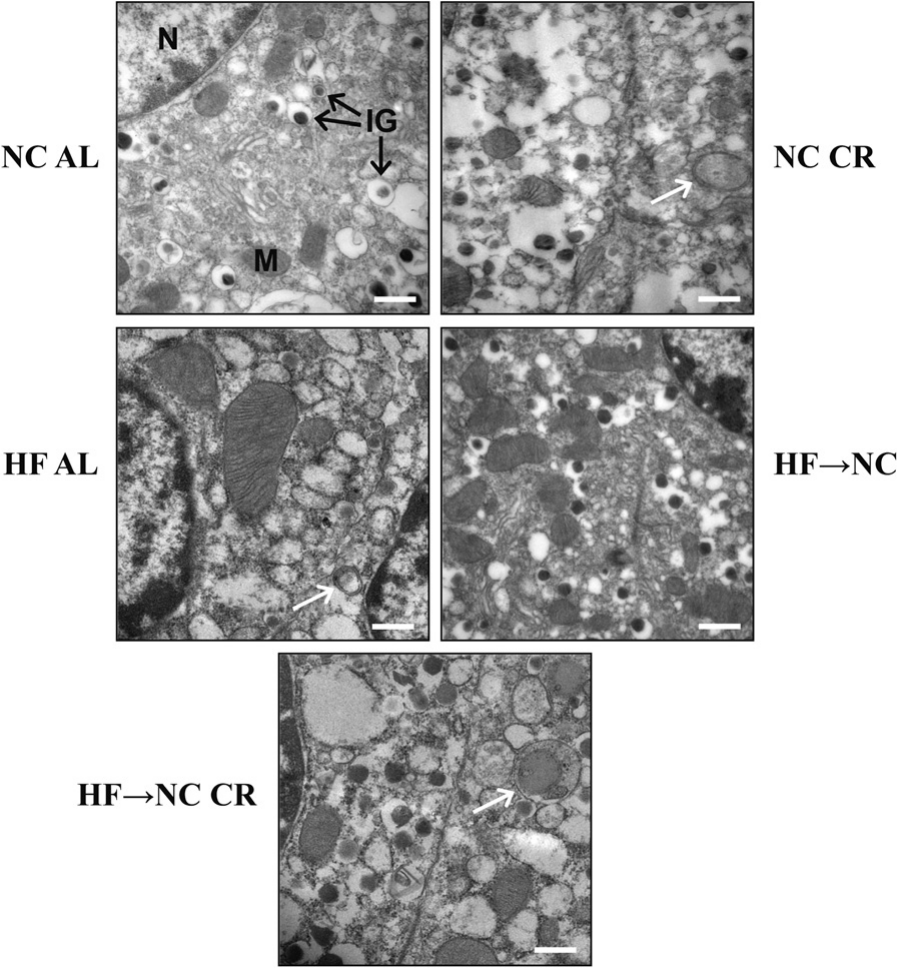
Transmission electron microscopic evaluation of autophagosomes in β-cells of mice. The pancreas tail tissues were collected for TEM analysis. Granular cytoplasm or degenerated organelles surrounded by double membrane representing autophagosomes (white arrows) were seen in β**-**cells of NC CR, HF AL and HF → NC CR mice. Images were representative of all the experiments (*n* = 3), scale bars: 0.5 μm. Abbreviations: TEM, transmission electron microscopy; N, nucleus; M, mitochondria; IG, insulin granules

**Fig. 7:**
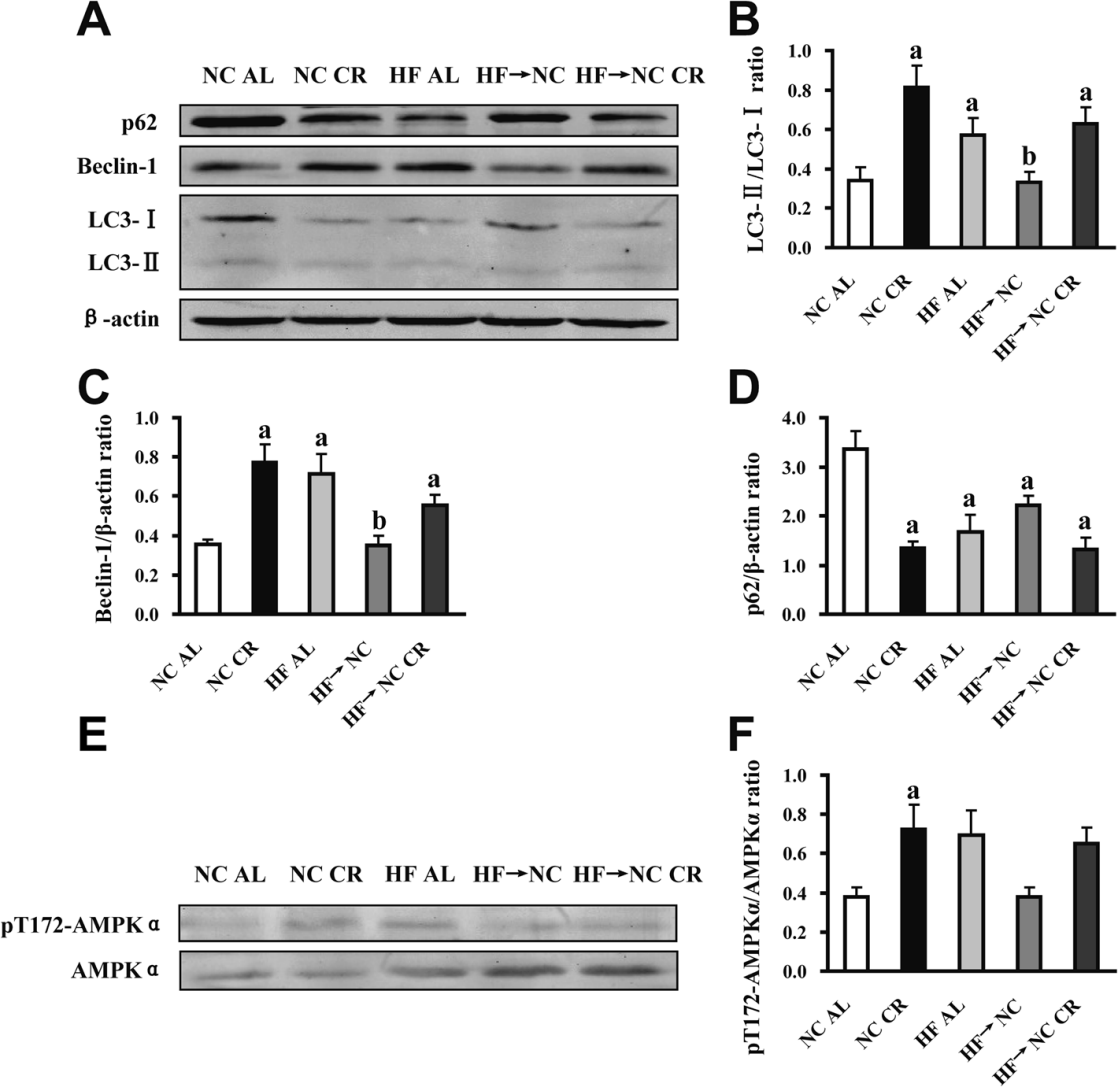
Effects of CR on the expression of autophagy markers and phosphorylated AMPK in islets. **a** LC3, beclin-1 and p62 protein levels in freshly isolated islets were determined by western blot, β-actin was used as an internal standard and representative blots were shown. **b** the ratio of LC3-II to LC3-I(*n* = 6). **c** and **d** the protein expressions of beclin-1 and p62 relative to β-actin (*n* = 6). **e** Representative blots of phosphorylated and total AMPKα protein expression in freshly isolated islets. **f** the ratio of pT172-AMPKα to total AMPKα (*n* = 6). Data were presented as means ± SE, *a* indicates *P* < 0.05 versus NC AL group; *b* indicates *P* < 0.05 versus HF AL group

Next, we detected the protein expressions of beclin-1, LC3II/LC3I (two specific markers for induction and formation of autophagosomes) and p62 (a marker being degraded by autophagy) in freshly isolated islets. Our findings showed significantly increased LC3II/LC3I ratio and beclin-1 level with remarkably decreased p62 accumulation in the NC CR, HF AL and HF → NC CR islets, indicating β-cell autophagy was activated in these three groups, but there were no differences in autophagy levels among these three groups. Autophagy levels in HF → NC islets were not different from those of the NC AL group (Fig. 7a-d). These results were further confirmed by TEM, which also showed an increased presence of autophagosomes in β-cells from the NC CR, HF AL and HF → NC CR groups (Fig. 6)

Because both CR groups showed activated β-cell autophagy, and autophagy activity was closely related with the energy metabolism level, we further examined the level of AMPK phosphorylation. The results showed that AMPKα phosphorylation was up-regulated in NC CR islets only, however, no significant differences were found among the other four groups (Fig. 7e and f).

## Discussion

In our study, chronic HF feeding was used to induce obesity in male C57BL/6 mice, which were then subjected to 3 weeks of switching to NC or 40 % CR to induce weight loss. Our findings demonstrated that moderate (40 %) CR to achieve normal weight could reverse β-cell dysfunction and insulin resistance, and restore glucose tolerance in DIO mice. The activation of β-cell autophagy might participate in this process.

After 12 weeks of HF feeding, HF AL mice became remarkably obese, and showed glucose intolerance and insulin resistance, accompanying by hypertrophic islets and increased β-cell area. Moreover, their early-phase insulin secretion tended to decrease and second-phase insulin secretion elevated significantly *in vivo.* This phenomenon is also commonly observed in patients with IGT (impaired glucose tolerance) or early-stage T2DM. *Ex vivo* isolated islets had increased insulin content and defective insulin secretion that manifested as enhanced basal insulin secretion and reduced high glucose-stimulated insulin secretion, suggesting β-cell dysfunction in HF mice was not due to insufficient insulin storage but to a defective secretion. These findings were consistent with previous studies [3, 20] that noted this secretory dysfunction was possibly caused by the reduced ATP/Ca^2+^ signaling. *In vitro* experiments eliminated the interference of confounding factors *in vivo*, such as insulin resistance, therefore it would be more accurate to reflect β-cell function. Furthermore, we observed activated β-cell autophagy in HF AL mice, this was in line with a previous study [13]. Several reasons could contribute to this phenomenon. Insulin resistance, free fatty acids (FFA), and misfolded proteins caused by endoplasmic reticulum stress are increased in obesity, and all of these factors can activate β-cell autophagy [21]. Obesity-induced activation of β-cell autophagy is considered as a compensatory response, providing cells with a safety mechanism to eliminate damaged mitochondria and/or other cellular components and to avoid cell apoptosis under insulin-resistant states and high FFA concentrations [13]. However, although enhanced β-cell autophagy played a protective role in HF AL mice, it was not strong enough to compensate for β-cell damage, therefore HF AL mice still had β-cell dysfunction.

It is reported that treating obesity by CR using a low fat diet attenuates the rebound weight gain compared with CR using an HF diet [22]. This was the basis of why we subjected DIO mice to CR with normal chow rather than CR with an HF diet. After 3 weeks of CR, HF → NC CR mice achieved normal weight, and fully recovered glucose tolerance, insulin sensitivity, insulin secretion and insulin content. Besides, increased organs weight, hyperlipemia, hypoadiponectinemia, and hypertrophic islets all returned to normal. Similarly, Lim et al. provided 11 newly diagnosed T2DM patients with VLCD of 600 kcal/day, and found that fasting blood glucose and hepatic insulin sensitivity fell to normal after 1 week of CR and β-cell function (insulin secretion rate and first-phase insulin response) increased towards normal over the 8 weeks of CR [7]. These results support our finding. Although the degree and duration of CR were different in these two studies, both studies confirmed β-cell dysfunction could be reversed by CR in obesity and the early stage of T2DM. However, it is very difficult to maintain this severe CR strategy. Another animal study [10] demonstrated that body weight and glucose tolerance were normalized after 3 weeks of 40 % CR in ob/ob mice, but circulating insulin levels remained high, implying insulin sensitivity was not restored. However, β-cell functions such as insulin synthesis and secretion were not explored in this study. Discrepancies in the restoration of insulin sensitivity between this previous study and our findings might be due to the difference in obese animal models used. Moreover, we further investigated the role of autophagy in this process and found that β-cell autophagy was activated in HF → NC CR mice. To our knowledge no study has yet reported the effect of CR on β-cell autophagy in obese mice, and there are only few studies that have reported autophagy is up-regulated in the heart and liver during CR in DIO mice or diabetic rats [23, 24]. Owing to the fact that autophagy can be activated under nutrient-poor conditions to maintain energy homeostasis [12, 13], we suppose that the sudden negative energy balance may contribute to the activation of β-cell autophagy in HF → NC CR mice. Considering the beneficial effect of autophagy on β-cells [13, 25], the activation of β-cell autophagy might contribute to the restoration of β-cell functions in HF → NC CR mice.

Switching from HF to NC without CR for 3 weeks normalized glucose tolerance, and reduced (but not normalized) body weight. It also showed unimproved early-phase insulin secretion *in vivo* and GSIS *in vitro*. Similarly, Agardh CD et al. demonstrated that switching from high-fat diet to low-fat diet for 4 weeks normalized glucose tolerance and reduced body weight in C57BL/6 J mice [26]. However, in contrast to our results, they showed improved early-phase insulin secretion in vivo. This might be due to that their treatments lasted for longer time. Additionally, many studies have indicated blood glucose levels return to normal earlier than body weight or insulin secretion [7, 9]. As HF → NC mice showed, although they achieved normoglycemia, defective insulin secretion still existed. Additionally, β-cell autophagy was not activated in HF → NC CR mice, which might be related with the unimproved insulin secretion of these mice. These findings suggest that besides normoglycemia, restoring β-cell function should also be regarded as the primary endpoint and target in intervention studies for obesity and T2DM.

In addition to applying CR on obese mice, we also evaluated the effects of CR on normal mice, and found decreased islet size and insulin content with unchanged GSIS, suggesting insulin storage but not insulin secretion was reduced. This was supported by a study that demonstrated CR potentiated amino acid-stimulated insulin secretion (AASIS) instead of GSIS [27]. Similar to that reported by Fok et al. [28], we also observed reduced blood glucose levels during IPGTT, although plasma insulin levels were normal, which might be explained by elevated insulin sensitivity in NC CR mice. Similar to the induced β-cell autophagy in 24-h fasted mice [12], we found β-cell autophagy was up-regulated in NC CR mice, and this might be due to acute energy depletion that was further confirmed by the presence of activated AMPK. However, both induction and inhibition of autophagy have been reported in starved INS-1 cells (a rat insulinoma-derived β-cell line), the variation of timing when depletion occurs might explain this inconsistency [13, 29]. As reported by previous studies, the up-regulation of β-cell autophagy can increase insulin secretion [13, 14, 29]. However, in our study, the insulin secretion of NC CR mice was comparable with that of NC AL group. The following reasons might explain this phenomenon. On the one hand, the activated β-cell autophagy in these mice could increase insulin secretion, but on the other hand, the fasting blood glucose levels of NC CR mice tended to decrease owing to their inadequate energy intake, and the decline of blood glucose levels would initiate the self-regulation mechanism of the body to reduce insulin secretion. So in general, the fasting plasma insulin levels of NC CR mice were kept in normal. In a word, short-term CR on normal mice had no significant effect on β-cell functions.

CR as an energy deficiency condition is closely related to increased intracellular AMP (adenosine monophosphate) level, which can lead to AMPK activation. And AMPK activation can directly induce autophagy [30]. We found AMPK was activated only in NC CR islets. However, the lack of AMPK activation in HF AL and HF → NC CR mice was probably due to the energy challenge not being severe enough to cause changes in AMP, and it implied the activation of β-cell autophagy in these mice was not through the AMPK pathway. It is reported that autophagy is a complex process regulated by multiple pathways including AMPK, mTOR, SIRT1, Bcl-2 and p53 [31, 32], therefore other pathways might be responsible for the activation of β-cell autophagy in HF AL and HF → NC CR mice.

## Conclusions

In conclusion, our study demonstrates for the first time that moderate (40 %) CR to achieve normal weight can reverse β-cell dysfunction and insulin resistance, and restore glucose homeostasis in DIO mice. Our study provides an encouraging sign that β-cell dysfunction is completely reversible in obesity and the early stage of T2DM. Furthermore, we propose that the activation of β-cell autophagy may play a role in this process, independent of AMPK activation. However, this hypothesis still needs to be confirmed, for example by using β cell-specific autophagy-deficient mice, and the responsible signaling pathways still require clarification.

## Abbreviations

DIO: Diet-induced obese
T2DM: Type 2 diabetes mellitus
VLCD: Very low–calorie diet
NC: Normal chow
HF: High-fat diet
AL: Ad libitum
CR: Calorie restriction
NEFA: Non-esterified fatty acids
IPGTT: Intraperitoneal glucose tolerance test
AUC: Area under the curve
HOMA-IR: Homeostasis model assessment of insulin resistance
HE: Hematoxylin and eosin
IHC: Immunohistochemistry
TEM: Transmission electron microscopy
GSIS: Glucose-stimulated insulin secretion
KRBH: Krebs-Ringer bicarbonate-HEPES buffer
IGT: Impaired glucose tolerance
AASIS: Amino acid-stimulated insulin secretion
AMP: Adenosine monophosphate
AMPK: Adenosine monophosphate-activated protein kinase
